# Spatially different annual cycles but similar haemosporidian infections in distant populations of collared sand martins

**DOI:** 10.1186/s40850-021-00071-z

**Published:** 2021-04-20

**Authors:** Steffen Hahn, Martins Briedis, Christos Barboutis, Raffaella Schmid, Martin Schulze, Nina Seifert, Tibor Szép, Tamara Emmenegger

**Affiliations:** 1 Department Bird Migration, Swiss Ornithological Institute, Seerose 1, CH-6204 Sempach, Switzerland; 2 Lab of Ornithology, Institute of Biology, University of Latvia, Salaspils, Latvia; 3 Antikythira Bird Observatory, Hellenic Ornithological Society/Birdlife Greece, Athens, Greece; 4 RANA, Agency for Ecology and Nature Conservation, Section Ornithology and Bird Conservation Merseburg e.V, Halle/Saale, Germany; 5 Michael Succow Foundation, Greifswald, Germany; 6 Institute of Environmental Sciences, University of Nyíregyháza, Nyíregyháza, Hungary; 7 Molecular Ecology and Evolution Lab, Lund University, Lund, Sweden

**Keywords:** Migration, Blood parasites, Parasitaemia, *Plasmodium*, *Haemoproteus*, Geolocation

## Abstract

**Background:**

Populations of long-distance migratory birds experience different environments and are consequently exposed to different parasites throughout their annual cycles. Though, specific whereabouts and accompanied host-parasite interactions remain unknown for most migratory passerines. Collared sand martins (*Riparia riparia*) breeding in the western Palaearctic spend the nonbreeding period in Africa, but it is not yet clear whether specific populations differ in overwintering locations and whether these also result in varying infections with vector-transmitted endoparasites.

**Results:**

Geolocator tracking revealed that collared sand martins from northern-central and central-eastern Europe migrate to distant nonbreeding sites in West Africa and the Lake Chad basin in central Africa, respectively. While the ranges of these populations were clearly separated throughout the year, they consistently spent up to 60% of the annual cycle in Africa. Ambient light recorded by geolocators further indicated unsheltered roosting during the nonbreeding season in Africa compared to the breeding season in Europe.

We found 5–26% prevalence of haemosporidian parasites in three breeding populations and one migratory passage population that was only sampled but not tracked. In total, we identified seven *Plasmodium* and nine *Haemoproteus* lineages (incl. two and seven new lineages, respectively), the latter presumably typical for swallows (*Hirundinae*) hosts. 99.5% of infections had a low intensity, typical for chronic infection stages, whereas three individuals (0.5%) showed high parasitaemia typical for acute infections during spring migration and breeding.

**Conclusions:**

Our study shows that blood parasite infections are common in several western Palaearctic breeding populations of collared sand martins who spent the nonbreeding season in West Africa and the lake Chad region. Due to long residency at the nonbreeding grounds blood parasite transmissions may mainly occur at host population-specific residences sites in Europe and Africa; the latter being likely facilitated by unsheltered roosting and thus high vulnerability to hematophagous insects. The rare cases of high parasitaemia during spring migration and breeding further indicates either relapses of chronic infection or primary infections which occurred shortly before migration and during breeding.

**Supplementary Information:**

The online version contains supplementary material available at 10.1186/s40850-021-00071-z.

## Background

Throughout the annual cycle, migratory birds and especially long-distance migrants reside at distant sites which often differ fundamentally in environmental conditions. Consequently, migrants are faced with region-specific availability of suitable habitats, food supply and predation risk. Moreover, they interact with local parasite and pathogen communities like hematophagous insects that are vectors for secondary (endo)parasites. Thus, populations which differ in migration routes and/or residence sites are expected to also differ in the parasite fauna they harbour [1].

Blood parasites (order Haemosporida) can be transmitted between birds when infected vectors take the blood meal. The avian host usually survives an acute infection stage, especially if host and parasite share a long evolutionary history [2]. Moreover, chronically infected hosts often show little or no changes in physiological capability like stress response, oxidative status and immune function [3] or in their aerobic capacity [4]. Consequently, migratory birds are able to transport endoparasites along the migration routes and thus connect distant ecosystems along the migratory flyway [5].

In migratory host species with large, continent-wide distributions, local breeding populations can have distinct nonbreeding areas with little or no overlap between populations [6–8], i.e., they show a high degree of migratory connectivity [9]. In such cases, host-parasite relationships might be specific to populations, especially if parasites are rarely transmitted between host populations due to little mixing of the vector fauna, limited breeding/nonbreeding site dispersal of hosts or differential timing of migration [10]. Consequently, this may result in population-specific parasite prevalence and diversity (see [11] for global assessment in a resident host species). Population-specific interactions between parasites and migratory hosts can differentially affect survival and annual reproduction [12, 13] and might have shaped the evolution of immune systems in passerines [14]. The populations of many migratory bird species have been declining rapidly during the last decades [15–17], mostly as a result of (human induced) changes in habitats and/or climate which affects resource availability at many sites occupied throughout the annual cycle [18, 19]. Additionally, rapid changes in environmental phenology like the advancing spring in the northern hemisphere can lead to temporal mismatches between food requirements and resource availability in local populations [20]. Such mismatches not only affect annual reproduction but may also influence individual condition and increase susceptibility for parasites [21]. Thus, knowledge about individual whereabouts throughout the annual cycle and interactions with parasites seems fundamental for a profound understanding of the ecology of a migratory host.

The collared sand martin (*Riparia riparia*, hereafter sand martin) is abundant and an obligate long-distance migrant with a Holarctic breeding range. The European breeding populations are estimated to 3.6 to 8 million breeding pairs [22] and all migrate to nonbreeding sites in sub-Saharan Africa. However, more specific information about migration routes are largely lacking and records of nonbreeding sites are mainly limited to the Senegal river delta [23]. The only available tracking data come from a Hungarian population, which migrates along the eastern Afro-Palearctic migratory flyway to the lake Chad region [24]. Ring recoveries from southern Europe and Africa further suggest a migratory divide with eastern breeding population migrating along the eastern Afro-Palearctic flyway and western populations along the western Afro-Palearctic flyway [23]. Such divergent migration can cause geographically distinct nonbreeding ranges [25]. Sand martins prefer to breed in cliffs such as steep banks along rivers; in central Europe the species also occupies man-made habitats in gravel pits or mining areas [26]. Most breeding and nonbreeding sites are related to wetland areas [23], which provide flying insects (e.g. mosquitoes) as abundant food supply. Thus, sand martins breeding and/or roosting in the vicinity of wetlands can be expected to serve as important hosts for vector-transmitted haemosporidian parasites. There is surprisingly little information on blood parasite infections in sand martins in general (i.e., [27]) with prevalence data ranging between 0 and 33% from spatially distant sites like Alaska [28, 29], Spain [30], Israel [31], Iraq [32], and Turkey [33]. The available data obviously do not cover the species’ entire range, samples sizes often remain very small and information on severity of infections, variation across years or full year perspectives is completely lacking. Thus, we aim to provide such basic data of the natural history by relating full annual cycles of sand martins breeding in northern central and in eastern central Europe with infection parameters of their haemosporidian parasites. This approach requires knowledge of year-round whereabouts of individuals and duration of main phases within the annual cycles, i.e., breeding period, nonbreeding residency and autumn and spring migrations. By sampling individuals from distant breeding colonies and at a migration passage site in Europe where inter-colonial movements across hundreds of kilometres is rare [34], we expect to find population specific prevalence of blood parasites especially if nonbreeding sites and migration routes differ between sampled populations. Additionally, if the transmission of blood parasites would occur primarily during the breeding season, we predict site-specific high parasitaemia in breeding birds corresponding to acute phase of infections [2]. Alternatively, if transmissions occur mainly outside the breeding period, infected birds should show high parasitaemia on spring migration and not during breeding. Further indications for seasonal infection probabilities can be deduced from stage-specific exposure to vectors and population-specific occurrence of blood parasite lineages.

## Results

### Annual cycle

**Fig. 1:**
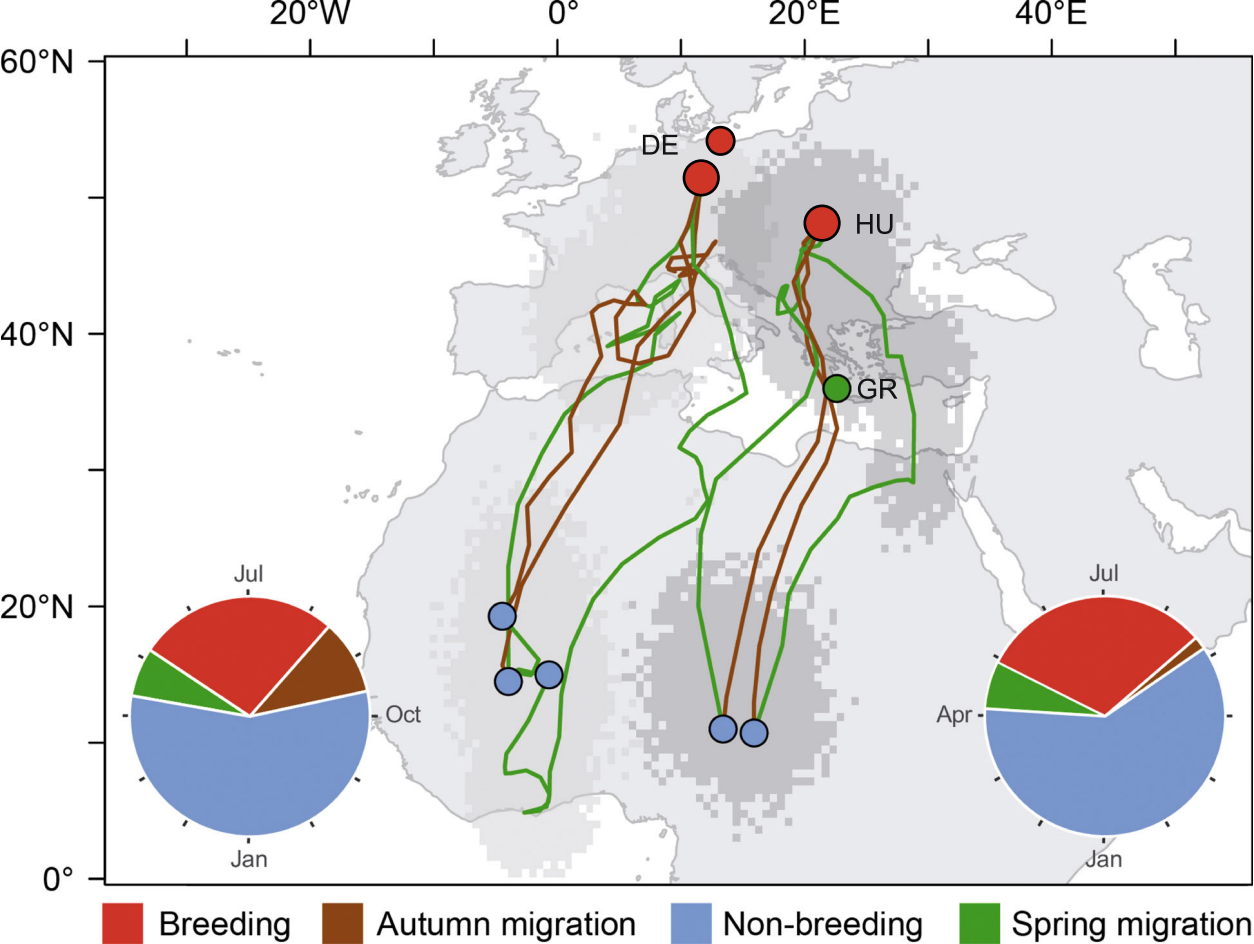
Annual cycle of four collared sand martins (*Riparia riparia*) breeding in north-central (DE) and south-central Europe (HU). The map gives the spatial organisation with autumn migration (brown) and spring migration (green) between the European breeding sites (red) and the sub-Saharan nonbreeding sites (blue). The underlying scatter indicates 95% credibility intervals of geolocation estimates (light grey: birds from Germany, dark grey: birds from Hungary). The green dot indicates the passage site in Greece (see main text). The insets give the temporal organisation with percentages of time for residency and migration periods (same period specific colours) and average arrival and departure for each respective period

Geolocator-tracked sand martins from the inland site in east Germany used the western Afro-Palearctic flyway by crossing the Alps and the central part of the Mediterranean Sea and subsequently flew across the western Sahara (Fig. 1). The birds spent the nonbreeding season in central West-Africa (Mali, Burkina Faso) with one bird itinerantly used three sites and finally ending up in Ghana/Ivory coast. In contrast, sand martins breeding in Hungary used the eastern Afro-Palearctic flyway via the Balkan peninsula, the eastern Mediterranean Sea and crossed the central part of the Sahara (Libya) to overwinter in the lake Chad basin, central Africa (Fig. 1). Both Hungarian birds remained stationary for the entire nonbreeding period and did not show site-itinerancy (similar to [24]). Thus, individuals originating from the same breeding sites overwintered in close vicinity to each other, and nonbreeding ranges of the populations were clearly separated by about 1300 km (Fig. 1).

Sand martins departed for autumn migration between August and September (Germany) and at the end of August (Hungary) and arrived at the nonbreeding grounds in September (supplementary material Table 1). Departure from nonbreeding grounds ranged from early to mid-April in Hungarian birds and from mid to end of April in German birds. They arrived at the European breeding grounds in the first half of May. Consequently, the tracked sand martins spent only one third (27–31%) of their annual cycle at their breeding sites in Europe, but 56–60% at the nonbreeding grounds in sub-Saharan Africa, and 2–10% on autumn and 6–7% on spring migration (Fig. 1, *inset*; supplementary material Table 1).

All tracked sand martins frequently roosted in cavities during the early breeding period with 29–64% and 33–53% of nights inside cavities for Hungarian and German birds, respectively. Such cavity overnights happened only occasionally during the pre-migration period (0–4% of nights) but were totally absent during the nonbreeding residence period and the autumn and spring migration periods.

### Infections with haemosporidian parasites

Infections with *Plasmodium* and/or *Haemoproteus* parasites were prevalent in all populations.

**Fig. 2:**
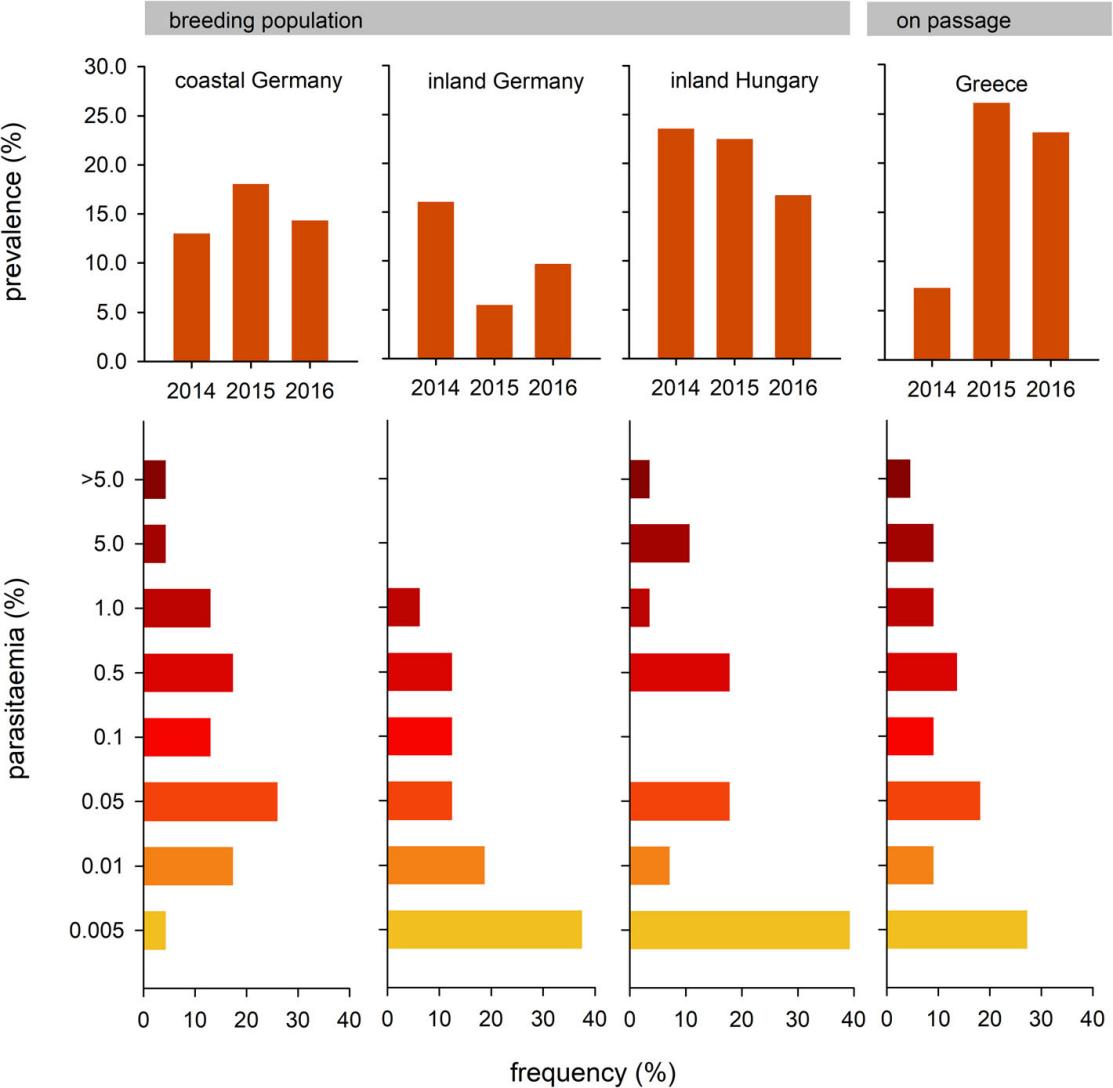
Site specific total prevalence (upper panel) and frequency of total parasitaemia of haemosporidian infections (lower panel) of collared sand martins (*Riparia riparia*) in northern-central and eastern-central Europe breeding in coastal and inland Germany and Hungary, and during migration in south-eastern Europe when passaging Greece. Parasitaemia, i.e., the strength of infection, represents the percentage of infected erythrocytes per sample

The site-specific annual prevalence ranged between 5.5 to 23.5% for the breeding colonies and 7.3 to 26.1% for the passaging birds (Fig. 2, upper panels). The total prevalence did not statistically differ between all sites (GLM, z values ranged between − 1.07 to 1.16, *p* > 0.24), and did not differ between study years (z = − 0.87, *p* = 0.38). Moreover, prevalence was not sex-specifically (GLM, z = 0.88, p = 0.38) with 11.1% in males (*n* = 144) and 17.1% in females (*n* = 281). The results did not change when excluding the 2015 Hungary subsample (see methods). Changes in total prevalence between years were not correlated across sites (Pearson correlation, all *p* > 0.17).

From 54 successfully sequenced samples, we identified seven *Plasmodium* lineages and nine *Haemoproteus* lineages, of which two and eight lineages had not been described before. The new *Plasmodium* lineages P-RIPRIP09 and P-RIPRIP 10 are closely related to the generalists P-SGS1 (*Plasmodium relictum*) and P-GRW06 lineages (*Plasmodium elongatum*); all seven new *Haemoproteus* lineages (H-RIPRIP01 to RIPRIP06 and H-RIPRIP08) were most similar to H-DELURB1 (*Haemoproteus hirundinis*), the new H-RIPRIP07 to H-DELURB2. Single infections with *Haemoproteus* lineages were equally frequent as single infections with *Plasmodium* lineages at the inland site in Germany and the passage site in Greece, while *Haemoproteus* lineages were 1.3 times more frequent at the coastal breeding site in Germany and 2.3 time more frequent at the Hungary breeding site. Of all 16 lineages, eight lineages were detected at two and more sites with H-RIPRIP 02 and P-SYBOR21 ubiquitously found at all four study sites (Table 1). The relative diversity of blood parasite lineages (number of lineages/numbers of infected hosts) was lowest in the coastal breeding site in Germany (0.41) and highest in Hungarian breeding birds (0.77; Table 1).

**Table 1:** Genetic lineages of haemosporidian parasites detected in collared sand martins (*Riparia riparia*) from breeding colonies in coastal and inland Germany (DE) and Hungary (HU), and from a spring migration passage site in Greece (GR). N gives sample size per site, and cells contain the number the number of infected hosts. Additionally, the nearest known lineage (for newly detected lineages), host orders and the potential region of infections based on information derived from the MalAvi database [35] are given. The relative parasite diversity gives the percentage of number of lineages per infected host individuals at a specific site.

		Breeding			Migration	Nearest known lineage		Region of Infection
Genus	Lineage	coast-DE (*n* = 17)	inland-DE (*n* = 10)	HU (*n* = 13)	GR (*n* = 14)		Host	
*Haemoproteus*	DELURB1			1			Hirundinidae	Not clear
	RIPRIP01*	5	1		1	DelUrb1	Hirundinidae	
	RIPRIP 02*	5	3	2	1	DelUrb1	Hirundinidae	
	RIPRIP 03*	1		3	1	DelUrb1	Hirundinidae	
	RIPRIP 04*	1		1		DelUrb1	Hirundinidae	
	RIPRIP 05*			1		DelUrb1	Hirundinidae	
	RIPRIP 06*			1	1	DelUrb1	Hirundinidae	
	RIPRIP 07*		1			DelUrb2	Hirundinidae	Not clear
	RIPRIP 08*			1		DelUrb1	Hirundinidae	
*Plasmodium*	GRW02	2	2		2		2 orders ^a^	Africa (Europe)
	GRW09		2				Passeriformes	Africa
	LAMPUR03	1					2 orders ^a^	Africa
	RIPRIP 09*				1	SGS1	11 orders ^a^	globally
	RIPRIP 10*			1		GRW06	3 orders ^a^	Europe/Africa
	SGS1			1	1		11 orders ^a^	globally
	SYBOR21	2	1	1	6		Passeriformes	Not clear
Relative diversity (%)		41.2	60	76.9	57.1			

* indicates newly detected lineages

^a^ including order Passeriformes

**Fig. 3:**
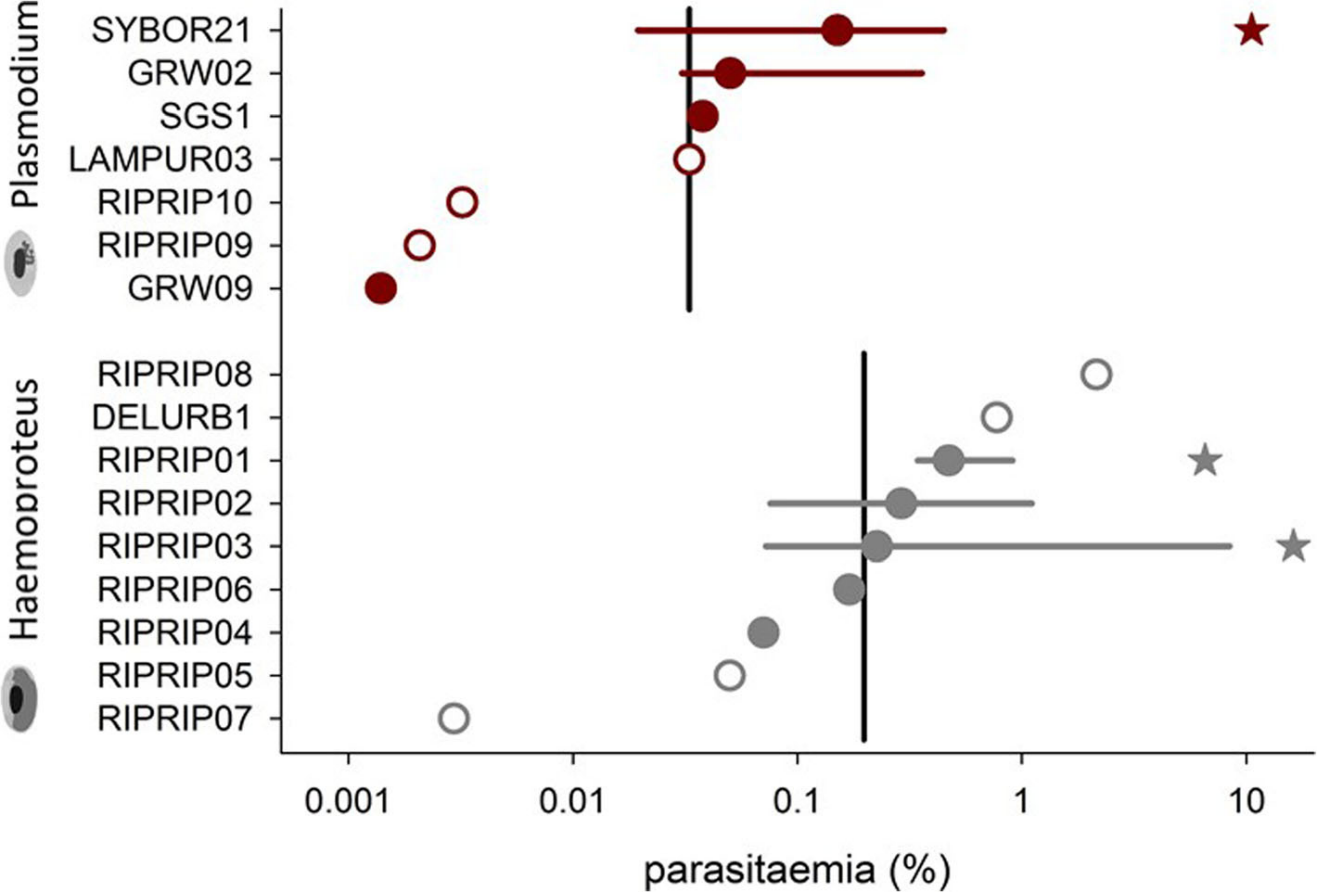
Parasitaemia of single infections with a specific *Plasmodium* (red) or *Haemoproteus* parasite lineage (grey) from collard Sand martins (*Riparia riparia*). Filled circles are medians (± 25/75 percentiles if *n* > 2), open circles give lineages with single observations. Black vertical lines indicate genus specific median parasitaemia. Median parasitaemia was genus specific and usually low, which is typical for chronic infections, except in three hosts (indicated with stars) infected with SYBOR21, RIPRIP01 and RIPRIP03 and maximum parasitaemia between 6.5 and 16.2%

Infection intensity (total parasitaemia) was generally low (Fig. 2, lower panel); the overall median parasitaemia (% infected erythrocytes) was 0.033% and site-specific medians ranged from 0.006% (inland site in eastern Germany) to 0.052% (Baltic coast site, Germany; for site specific differences, log10 transformed: H_3,88_ = 6.36, *p* = 0.10). However, we found three individuals with exceptionally high parasitaemia: one bird from the Baltic coast population with 6.55% parasitaemia from a single infection with H-RIPRIP01, one individual during passage in Greece with 10.6% parasitaemia from a single infection with P-SYBOR21, and finally one breeding bird in Hungary with 16.2% parasitaemia from a single infection with H-RIPRIP03 (Fig. 3). The average parasitaemia with *Plasmodium* lineages was significantly lower than those with *Haemoproteus* lineages (single infections; log10 transformed data: t_52_ = 3.38, *p* = 0.001; Fig. 3).

## Discussion

Using geolocators, we identified separate but parallel migration pattern of sand martins from distant breeding populations in northern central (Germany) and eastern central Europe (Hungary). We acknowledge that our results and conclusions on migration pattern originate from a relatively small sample of females. Although we assume that our sample of individuals is representative for the respective population, we await results of more extensive tracking data that confirm (or modify) our findings.

We found no overlap in nonbreeding residence sites of tracked females of these populations. Ring recoveries for population-specific African nonbreeding ranges outside the Senegal Delta in West Africa are very rare [23] and cannot document site itinerancy. Moreover, information about sex-specific nonbreeding ranges or divergent behaviour during the nonbreeding period in Africa is not (yet) available. Assuming the migration pattern of our tracked females is representative for both females and males of the respective study population, sand martins are likely to use delimited, population-specific regions during the nonbreeding period (see also [24]) indicating high degree of migratory connectivity. In Palaearctic Barn swallows (*Hirundo rustica*) with comparable degree of migratory connectivity, residence sites during the nonbreeding periods did not differ between sexes [36]. Presuming a similar pattern, male and female sand martins of a focal population should overwinter in the same region and its rather unlikely that they disperse over wider distances in sub-Saharan Africa (contrary to assumption in [37]). Strong migratory connectivity and parallel migration in populations from the western Palaearctic would result in nonbreeding distributions that mirror the longitudinal distribution of the breeding sites [25]. Therefore, neighbouring populations might experience more similar environmental conditions in both seasons than distant populations. If conditions at nonbreeding sites affect demographic rates to a large degree [23, 38] we would also expect similar population trajectories in neighbouring populations.

Our tracked birds spent more than half of the annual cycle at the nonbreeding grounds in Africa, only one fourth to one third of the year at the breeding grounds, and much less time on migration (Fig. 1). These estimates seem robust, as observational data between 2007 and 2019 at the two breeding sites in Germany confirm similar ranges for first arrival (range: 30 March – 07 May) and departure dates (range: 07 August – 09 October, unpubl data N.Seifert, M.Schulze). Moreover, the only currently published tracking of sand martins also showed that birds spent 57% of the annual cycle at the nonbreeding residences in Africa [24]. The light data records from geolocators further indicated that sand martins changed roosting behaviour within the annual cycle: on migration and during the nonbreeding period they usually roost outside from cavities (this study), often in reed beds and shrubs along water bodies [23, 26] – habitats typically associated with high densities of haematophagous insects. In contrast, during breeding, especially during the incubation period adults of both sexes regularly stay overnight in breeding cavities ([26, 39], only for females: this study). A nocturnal exposure to the open environment may increase the risk of being attacked by crepuscular and nocturnal haematophagous insects, which are the vectors of avian blood parasites [2, 40].

The spatial and temporal organisation of the annual cycle of hosts is a key component for host parasite interactions, especially in migratory species. Travelling between distant sites of residency within short periods of time decreases the risk of infection during migration. Consequently, sand martins likely experience seasonally variable risks of parasite transmission: high risk during the long nonbreeding residence period, intermediate and low during breeding and migration periods, respectively. Thus, conditions during periods of residency at a specific site will determine the likelihood of parasite infections. Based on the timing of the annual cycle (Fig. 1) and specific roosting behaviour our tracking sub-study suggest a lower risk of blood parasite transmission at the mid-latitude breeding grounds compared to the nonbreeding regions at low latitudes with high pathogen richness (i.e. [14]) .

Quantifying specific blood parasites transmissions throughout the annul cycle is a difficult task, especially in long-distance migrants with nonbreeding sites anywhere in Africa. Thus, infections at sand martins’ breeding sites might be detectable in future studies by repeatedly testing local breeders after arrival and before departure and fledged juveniles before departure.

Most adult birds captured during breeding - weeks after departure from the nonbreeding sites - showed low infection intensities (Fig. 2) which are indicative of chronic infection stages.

The predominance of low parasitaemia in our sample might be slightly biased as we captured (active) individuals by mist-netting and thus may have missed inactive birds during the acute phase of infection [41]. Though, we found three exceptional cases out of 569 checked individuals: two birds sampled during the breeding periods and one sampled on spring migration showed high parasitaemia usually seen during the acute infection stage. In the breeders we identified *Haemoproteus* parasite lineages, a genus which often shows relatively high parasitaemia during a chronic infection stage [42]. Unfortunately, we cannot unambiguously infer the origin of the infection as the parasite lineages (H-RIPRIP01 and H-RIPRIP03) had not been detected before. The third case, the bird on spring migration, had parasitaemia of almost 11% caused by *Plasmodium* lineage P-SYBOR21 (Fig. 3). An acute phase parasitaemia usually develops a few days post-transmission and lasts a week or longer depending on the parasite lineage [2, 43]. Thus, the case of an active migrant carrying a strong *Plasmodium* infection indicates either transmission in Africa followed by immediate migration towards the breeding site or, similarly likely, a relapse of a formerly chronic infection just before or during the migration towards the breeding site. In either case, the bird was able to fly across the Sahara Desert and the Mediterranean Sea irrespective of its potentially challenged metabolism and immune system [44]. However, based on the organisation of the annual cycle (Fig. 1) and the seasonally different roosting behaviour we expect that many infections in our studied sand martin populations are transmitted in Africa, which does not exclude local transmission during the breeding, like reported for a population at the Iberian peninsula [30]. Thus, ranking the significance of several transmission areas of blood parasites for this host species can only be tentative due to the incomplete coverage in monitoring the degree of parasitism and the blood parasite lineages (Table 2).

**Table 2:** Site specific numbers of deployed and retrieved geolocators as well as the number of obtained blood samples and blood smears during the study years from 2014 to 2016. Sites were breeding colonies at coastal and inland Germany (DE), and inland Hungary (HU) and a migration passage site at a Greek island in the Mediterranean Sea (GR)

	Geolocator (deployed/retrieved)			Blood samples		
Site	2014	2015	2016	2014	2015	2016
DE-coast	25 / -	25 / 0	- / 0	54	50	49
DE-inland	25 / -	25 / 0	- / 2	50	54	52
HU- inland	25 / -	25 / 0	- / 2	51	49^a^	30
GR				55	23	52

^a^blood smears only

Our combined approach of host tracking and blood parasite monitoring revealed differential annual prevalence of blood parasites in local breeding populations indicating population-specific host-parasite interactions. Dispersal between the breeding colonies and therefore an exchange of infected sand martins is more likely to occur between nearby colonies than between distant colonies, because ca. 90% of all inter-colonial movements occurred at distances < 100 km [34]. If transmission would indeed mainly occur at sub-Saharan nonbreeding sites, we would expect to find large differences in blood parasite diversity in those host populations which have fundamentally different nonbreeding regions, like the Sahelian zone for the central European populations (this study) and south or eastern Africa (presumed for eastern Palaearctic breeding populations).

## Conclusion

Our study reveals differential migration routes and nonbreeding sites but similar temporal organisation of the annual cycle in sand martins from northern-central and central-eastern European breeding populations. Western Palaearctic Sand martins are commonly parasitized by haemosporidians with annual prevalence ranging between 5 to 26%. The low parasitaemia detected during breeding implies that most blood parasites might be mainly transmitted at host population-specific nonbreeding sites in Africa. The transmission is likely facilitated by unsheltered roosting and thus high vulnerability to hematophagous insects. The rare cases of high parasitaemia in birds tested during spring migration and breeding further indicates either relapses of chronic infection or a primary infection shortly before migration and during breeding. Due to considerable between-year variation in prevalence and the numerous new genetic parasite lineages our study cannot finally conclude if population-specific migration and the resultant different nonbreeding regions cause differential blood parasitism in sand martins.

## Methods

We captured adult sand martins at three breeding sites and at one spring migration passage site in northern-central, eastern-central, and south-eastern Europe. Breeding colonies were located at a brackish lagoon of the Baltic Sea coast in Germany (54° 8′N, 13°25′E, two small colonies ca. 8 km apart), at an inland site in an agricultural landscape in Saxony-Anhalt in eastern Germany (51°27′N, 11°40′E), and in a riverine wetland of the Tisza river in eastern Hungary (48°11′N, 21°28′ E). The passage site was situated on Antikythera island (GR, 35°52′N 23°18′E) in the eastern Mediterranean Sea; the island does not house breeding colonies of sand martins. Minimum distances between breeding sites were 320 km within Germany, 795 km for Germany-Hungary and 1′375 km from the Hungarian breeding site to the passage site at the Greek island.

Adult birds were captured using mist-nets either during the chick rearing periods in June/beginning of July (breeding sites) or during spring migration in May (passage site) in three consecutive years from 2014 to 2016. Birds were ringed, measured (body mass, wing, and primary length), and sexed by cloacal protuberance and brood patch.

### Tracking sand martins’ annual cycle

We used light-level geolocators to track individual migrations from breeding to nonbreeding sites and back. To this end, we equipped 25 adult females at each breeding site and year (2014, 2015) with SOI-GDL2.0 geolocators (Swiss Ornithological Institute) (Table 2). These geolocators weighed on averaged 0.62 g equalling 4.6% of adult body mass at capture. We only retrieved four functional geolocators, all from birds tagged in 2015 from the inland colonies in Germany and Hungary (two geolocators per site) (supplementary material Table 1). We failed to retrieve any geolocator from the site at the Baltic Sea coast.

We analysed the light intensity data with a threshold approach following the guidelines described in [45] (also see: https://geolocationmanual.vogelwarte.ch/). First, we derived sunrise and sunset times from the raw light recordings in R-package *TwGeos* [46] using a light intensity threshold of one arbitrary unit. Second, we calibrated the recorded twilight events against the actual sunrise and sunset times at the respective breeding (tagging) locations of the birds prior to the start of the autumn migration (i.e., in-habitat calibration, [47]). Further, to distinguished between stationary and movement periods we applied the *invChanges* function from the R-package *GeoLight* v2.01 [48] to identify changes in sunrise and sunset times. The minimum length of a stationary period was set to 2 days. Finally, we used the R-package *SGAT* [49] to reconstruct migration tracks by applying the grouped model in combination with a spatial mask that allowed stationary sites to be located only on land. The twilight error distribution was assumed to follow Gamma distribution (parameters as inferred from the in-habitat calibration) and the movement model was assumed to follow Gaussian distribution (shape = 10, scale = 0.2) with the highest probability of travel speeds between 40 and 60 km h^− 1^ during the movement phases. The beginning and the end of the tracks were fixed to the respective breeding locations. To initiate the model, we first ran a *modifiedGamma* model with relaxed assumptions for 1000 iterations, and then tuned the resulting model three times (300 iterations each) with final assumptions/priors. As a final step, we run the model for 2000 iterations to ensure convergence and extracted median location estimates and their 95% credibility intervals.

We determined four main periods within the annual cycle: the breeding and the nonbreeding periods are times when birds are resident, reproduce or moult and engage in daily foraging flights, while the autumn and the spring migration period encompass movements and stopover times when birds travel between the two sites of residence. Transitions between these periods, departure and arrival dates were derived from the results of *invChanges* function analysis (see above), main residence periods/sites are defined to last longer than 4 weeks. Finally tracks and positions were mapped using R package *rworldmaps* [50].

### Daily exposure to potential vectors

Many vectors of haemosporidian parasites like mosquitoes and midges (but not louse flies) are particularly active during twilight periods [51]. A potential host might reduce its infection risk by hiding in shelters during peak times of vector activity. Many cavity breeders like swallows are well known to roost in cavities during breeding [26], and thus being less exposed to vectors during the twilight periods. Cavity roosting during the nonbreeding periods is not well studied. We quantified cavity roosting of geolocator birds during twilight periods throughout the annual cycle by counting non-natural pattern of sunrises and sunsets when ambient light intensity records abruptly changed e.g. when leaving a cavity after sunrise or entering a cavity before sunset [24]. Geolocator data provided full records during the nonbreeding residence periods and the migration periods, but incomplete data for the breeding period due to delayed start of light measurements (July) in the year of deployment and the immediate removal of the geolocator after arrival in the subsequent year. Thus, cavity use estimates during breeding periods are minimum values encompassing early incubation and late chick rearing times.

### Infections with haemosporidian parasites

We investigated infections with haemosporidians of the genera *Plasmodium* and *Haemoproteus* at all four sites in all 3 years (Table 2). We collected blood samples from a total of 569 adult birds (i.e., second year birds or older) with 153 samples (70 males, 72 females and 11 birds of unknown sex) from the Baltic Sea coast colonies (Germany), 156 samples (59 males, 96 females, one of unknown sex) from the inland colony in Germany, 130 individuals (15 males, 113 females, two of unknown sex) from the inland colony in Hungary and 130 samples from unsexed individuals on passage in Greece (Table 2). The breeding origins of these passaging migrants were not known but very likely being situated in eastern Europe (e.g. [24]).

We sampled about 30 μl peripheral blood by puncturing the brachial vein with a sterile needle and collecting the whole blood with a heparinized capillary tube to make blood smears (fixed in methanol within 24 h and later stained with Giemsa solution). Remaining blood was immediately stored in SET buffer (0.015 M NaCl, 0.05 M Tris, 0.001 M EDTA, adjusted to pH 8 with NaOH) for later genetic analysis (see also [52] for method details). Blood samples for genetic analysis were not available for the 2015 sub-sample from Hungary (Table 2), therefore the infection status of these birds was determined by classical microscopy of Giemsa-stained blood smears (see below for details on microscopy).

We detected infections with *Haemoproteus* sp. and *Plasmodium* sp. genetically by PCR (except for the sub-sample from Hungary in 2015). To this end, we extracted DNA (DNeasy blood and tissue kit, Qiagen) and performed a standard nested PCR (primer pairs: nested 1 = HaemNF1 + HaemNR3; nested 2 = HaemF + HaemR2; developed by Hellgren et al. [53]). The PCR products were visualised in 2% agarose gel stained with GelRed (Biotium Inc.). Positive samples showed a clear band at approximately 500 bp, samples with ambiguous PCR results (weak bands) were rerun and additionally screened by microscopy (see below) to reduce risk of false negative results. We calculated the total blood parasite prevalence as the percentage of infected individuals per site, study year and sex (for breeding sites only). For prevalence we did not discriminate between single and mixed infections as well as between blood parasite lineages or genera.

We quantified the intensity of individual infections, i.e., parasitaemia as the percentage of infected erythrocytes, and the infection status for the 2015 subsample from Hungary (see above) by classical microscopy of Giemsa-stained blood smears. We counted infected erythrocytes in 100 microscopic fields with 1000× magnification (Primo Star, Carl Zeiss AG). The total number of inspected erythrocytes was extrapolated from a known number of erythrocytes of five pictures taken by a Zeiss AxioCam ERc5s camera following [54]. In case we found no infected erythrocyte or only extracellular parasite stages in a blood smear of a PCR-positive sample (*n* = 6), we set the parasitaemia to half the minimum detectable intensity (i.e. 0.5 infected erythrocytes / total number of inspected erythrocytes; see [4]).

Finally, we determined the genus *Plasmodium* or/and *Haemoproteus* and the genetic lineages of the respective parasites. We purified the PCR products of parasite-positive and unclear samples (still weak bands after repetition), sequenced them once with the forward (HaemF) and once with the backward primer (HaemR2). For each sequencing run (following the manufacturers protocols of the sequencing kit ABI BigDye® Terminator v3.1 Cycle, Applied Biosystems) the fluorescent-labelled fragments were analysed by electrophoresis in a DNA analyser (ABI 3730xl, Applied Biosystems). All sequences were subsequently checked, edited and merged in BioEdit [55]. The consensus sequences were compared to known lineages from the MalAvi database [35]; accessed on 19/04/19) by performing a multi-sequence analysis (R package *msa* [56]; see statistics). From the total of 103 sequenced samples, 29 samples repeatedly showed no signal and, if we did not find any parasite by microscopy, we considered a sample as ‘uninfected’. For another 20 sequences we could only verify infection status but not lineages due to low DNA quality and/or mixed infections. Ultimately, we obtained 54 reads of full length (479 bp). While 22 full-length sequences matched 100% with lineages registered on MalAvi, 32 of the full-length sequences did match up to 99% to known lineages and are therefore described as new lineages (for lineage archiving see: Availability of data and materials).

### Statistics

We compared prevalence data between populations, years and sexes by fitting a Generalised Linear Model (GLM; function glm of the R package lme4; R Core Team, 2019: https://cran.r-project.org) with infection status as the dependent variable (family = binomial) as well as population, year and sex as independent variables. Annual changes of prevalence between sites were tested using Pearson’s correlations. Further, we compared infection intensity (parasitaemia) between sites using Kruskal-Wallis one-way analysis of variance on ranks tests for non-normally distributed data. Statistical tests were run in R (https://www.r-project.org/) and SigmaPlot11.

## Supplementary Information

**Additional file 1: Supplementary material Table 1.** Information of four geolocator-tracked female Sand martins from Germany and Hungary. Details include capture and recapture dates, body masses as well as the timing of key events within the annual cycle. For details on methods see main text. All tracking data are available on https://www.movebank.org; project #1292418712 (Germany) and project #1294793428 (Hungary).

## Data Availability

Tracking data are available on https://www.movebank.org; project #1294793428 (birds from Hungary) and #1292418712 (from Germany). Data of blood parasites are archived at MalAvi database (http://130.235.244.92/Malavi/) (see: [35]). Newly discovered blood parasite cytb lineages have been registered in GenBank (https://www.ncbi.nlm.nih.gov/genbank/) (accession no. MT419895 - MT419904).
