# Supplementary material for: Spatially different annual cycles but similar haemosporidian infections in distant populations of collared sand martins
Source: BMC Zool. 2021 Apr 20;6:6. doi: 10.1186/s40850-021-00071-z (PMC10127412; doi:10.1186/s40850-021-00071-z)
Supplement: Supplementary file 1 — Additional file 1: Supplementary material Table 1. Information of four geolocator-tracked female Sand martins from Germany and Hungary. Details include capture and recapture dates, body masses as well as the timing of key events within the annual cycle. For details on methods see main text. All tracking data are available on https://www.movebank.org; project #1292418712 (Germany) and project #1294793428 (Hungary). [file 40850_2021_71_MOESM1_ESM.docx]

Supplementary material

Table 1: Information of four geolocator-tracked female Sand martins from Germany and Hungary. Details include capture and recapture dates, body masses as well as the timing of key events within the annual cycle. For details on methods see main text. All tracking data are available on <https://www.movebank.org>; project #1292418712 (Germany) and project #1294793428 (Hungary).

| Site | Germany | | Hungary | |
| --- | --- | --- | --- | --- |
| Ring ID | ZG53012 | ZG53036 | W320953 | W261031 |
| Date capture | 30.05.2015 | 31.05.2015 | 19.06.2015 | 17.06.2015 |
| Date recapture | 06.06.2016 | 07.06.2016 | 26.05.2016 | 19.05.2016 |
| Bod mass (g) cap/recap | 15.1/15.3 | 14.9/14.1 | 12.2/13.1 | 13.9/14.4 |
| Departure BS | 07 Aug | 06 Sept | 29 Aug | 01 Sept |
| Arrival NBS | 22 Sept | 04 Oct | 07 Sept | 06 Sept |
| Departure NBS | 15 Apr | 24 Apr | 08 Apr | 18 Apr |
| Arrival BS | 21 May | 07 May | 10 May | 05 May |
| Duration (d) AM/NBS/SM | 47/207/37 | 29/204/14 | 10/215/33 | 6/226/18 |

BS – breeding site; NBS – nonbreeding site; AM – autumn migration; SM – spring migration
